# Rapid Directional Change Degrades GPS Distance Measurement Validity during Intermittent Intensity Running

**DOI:** 10.1371/journal.pone.0093693

**Published:** 2014-04-14

**Authors:** Jonathan C. Rawstorn, Ralph Maddison, Ajmol Ali, Andrew Foskett, Nicholas Gant

**Affiliations:** 1 Exercise Metabolism Laboratory, Department of Sport and Exercise Science, The University of Auckland, Auckland, New Zealand; 2 National Institute for Health Innovation, School of Population Health, The University of Auckland, Auckland, New Zealand; 3 School of Sport and Exercise, Massey University, Auckland, New Zealand; University of Bath, United Kingdom

## Abstract

Use of the Global Positioning System (GPS) for quantifying athletic performance is common in many team sports. The effect of running velocity on measurement validity is well established, but the influence of rapid directional change is not well understood in team sport applications. This effect was systematically evaluated using multidirectional and curvilinear adaptations of a validated soccer simulation protocol that maintained identical velocity profiles. Team sport athletes completed 90 min trials of the Loughborough Intermittent Shuttle-running Test movement pattern on curvilinear, and multidirectional shuttle running tracks while wearing a 5 Hz (with interpolated 15 Hz output) GPS device. Reference total distance (13 200 m) was systematically over- and underestimated during curvilinear (2.61±0.80%) and shuttle (−3.17±2.46%) trials, respectively. Within-epoch measurement uncertainty dispersion was widest during the shuttle trial, particularly during the jog and run phases. Relative measurement reliability was excellent during both trials (Curvilinear r = 1.00, slope = 1.03, ICC = 1.00; Shuttle r = 0.99, slope = 0.97, ICC = 0.99). Absolute measurement reliability was superior during the curvilinear trial (Curvilinear SEM = 0 m, CV = 2.16%, LOA ± 223 m; Shuttle SEM = 119 m, CV = 2.44%, LOA ± 453 m). Rapid directional change degrades the accuracy and absolute reliability of GPS distance measurement, and caution is recommended when using GPS to quantify rapid multidirectional movement patterns.

## Introduction

Use of Global Positioning System (GPS) technology as a performance analysis tool is increasingly common in a number of team sports [1]–[15]. Several studies have been dedicated to assessing the validity of GPS devices for this purpose, although newer devices have also been used without prior validation [13]–[15]. Despite considerable variation in experimental design previous investigations report small relative distance and velocity measurement uncertainties, and prevailing conclusions support the use of GPS devices during team sport activities [16]–[25].

While GPS devices appear acceptably valid for quantifying performance across entire bouts of exercise, sport scientists, coaches and governing bodies have shown particular interest in quantifying the high intensity activity demands of match-play [6], [7], [10], [26], [27]. The utility of GPS for monitoring performance is contingent on accurate and reliable measurement of these activities, which play a critical role in determining athletes' physiological load, and competitive match outcomes [28]–[31]. Sprinting and rapid acceleration are consistently associated with increased GPS measurement uncertainty, particularly over short distances [16], [19], [22], [23], [32], [33]. Thus it appears likely that GPS devices underestimate some movement patterns that are of critical importance during training and match-play.

While the effect of running velocity on GPS measurement validity is well established little attention has been directed toward determining the effect of rapid directional change. As athletes may execute ∼550–730 turning movements during match-play [28], [34] it is important to determine whether rapid directional change affects GPS measurement validity. One investigation has compared GPS measurement validity during linear and non-linear running, and rapid directional change was associated with reduced measurement validity [18]. However, differing self-selected velocity profiles between the linear and non-linear protocols make it difficult to determine whether this effect was caused by differing directional demands, velocity demands, or a combination of both. Thus there remains a need to systematically determine whether rapid directional change effects GPS distance measurement validity during exercise protocols with equivalent velocity profiles, but differing directional demands.

The Loughborough Intermittent Shuttle-running Test (LIST) is a precisely controlled intermittent intensity shuttle-running protocol designed to simulate the activity patterns of soccer [35]. The LIST is representative of the total distance, number of sprints and number of turns common to match-play, and induces similar physiological responses [35], [36]. Furthermore, precise control over velocity throughout the LIST facilitates systematic evaluation of the effect rapid directional change exerts on GPS measurement validity by allowing modification of directional demands without altering the velocity profile.

Many early investigations evaluated GPS devices featuring a 1 Hz sampling frequency, yet more recent devices with faster sampling frequencies have demonstrated improved measurement validity during linear and multidirectional running [19], [22], [33]. A novel device comprising a 5 Hz GPS microcontroller and an interpolation algorithm that outputs positional data at a 15 Hz frequency was recently utilised to investigate the movement demands of soccer, rugby union and rugby sevens [13]–[15]; however, the distance measurement validity of this device has yet to be evaluated. Therefore, the aim of this study was to systematically assess the effect of rapid directional change on the distance measurement validity of a previously untested GPS device. It was hypothesised that rapid directional change would reduce distance measurement validity.

## Materials and Methods

### Ethics statement

This study received approval from the University of Auckland Human Participants Ethics Committee. All volunteers provided written informed consent prior to participation.

### Participants

Six amateur club and provincial level team sport athletes (age = 24.1±1.6 y, body mass = 72.56±10.33 kg, height = 1.79±0.09 m, 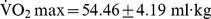) volunteered to participate in this study.

### Experimental protocol

During a preliminary trial participants completed a multi-stage fitness test to estimate maximal oxygen consumption (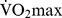), and determine running velocities for each phase of the LIST, as previously described [37]. Participants also completed a 15 min bout of the LIST while wearing the GPS device to familiarise themselves with the movement pattern, and device operation.

Participants completed two experimental trials in random order, within 7 days. Participants completed ≈90 min of the LIST movement pattern on shuttle or curvilinear running tracks. The movement pattern comprised sequential walk (60 m, velocity = 1.54 m·s^−1^), sprint (15 m, maximal velocity; 5 m deceleration), run (60 m, velocity eliciting 95% 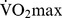) and jog (60 m, velocity eliciting 55% 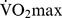) phases, as previously described [35], [38]. This cycle was repeated 11 times (≈15 min) during each of six exercise blocks. Exercise blocks were separated by 3 min rest periods. Reference cycle, block and trial distances were 200 m, 2 200 m, and 13 200 m, respectively. Identical regulation of movement velocity during both trials, via standardised auditory commands, ensured movement demands differed only in the presence (shuttle) or absence (curvilinear) of rapid directional change. Moreover, velocity regulation controlled for potential disruptive effects of environment or other extraneous variables on participants' performance and, therefore, on reference measures.

**Figure 1:**
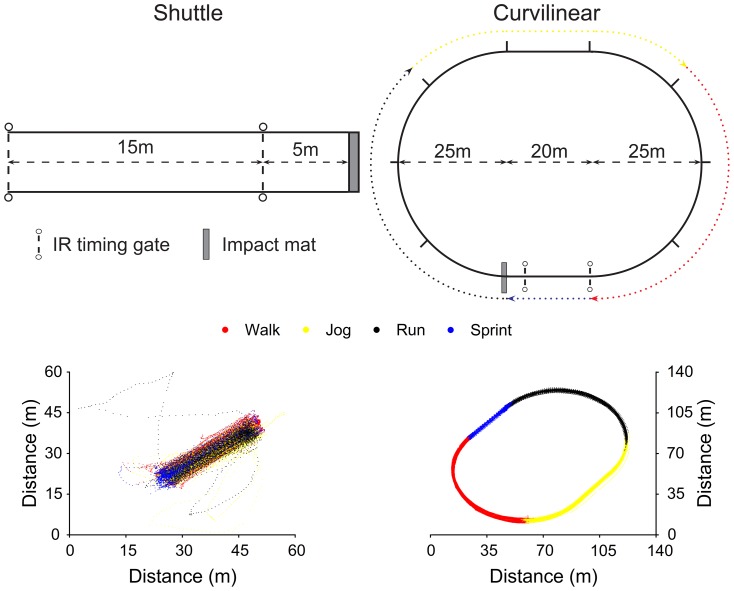
Schematic and satellite representations of shuttle and curvilinear running tracks. Satellite representations comprise typical positional data from one shuttle and curvilinear trial. IR = Infrared.

Schematic and satellite representations of the shuttle and curvilinear protocols are displayed in Figure 1. Briefly, the shuttle protocol was completed on a marked 20 m shuttle-running track similar to that described by Nicholas et al. [35], and the curvilinear protocol was completed on a marked oval track on a level athletic playing surface. The curvilinear track was designed such that one lap represented one 200 m movement cycle, the 20 m sprint followed a linear path and turn radii (25.5 m) were optimised to minimise rapid directional change. Markings at 20 m intervals along the curvilinear track facilitated adherence to velocity regulation commands as per the shuttle protocol. A foam impact mat precluded excess displacement following sprint phases during both trials. The mat was temporarily withdrawn after impact during the curvilinear trial to prevent participant deviation from the marked track. Participants who chose not to use the impact mat to aid deceleration were instructed to proceed to the mat after deceleration to ensure the correct distance was covered. A researcher monitored adherence to marked running tracks in order to prevent deviation. The shuttle and curvilinear tracks were located away from large buildings to minimise multi-pathing error and ensure clear line of sight to orbiting satellites. Track lengths were measured with a calibrated surveyor's wheel.

### GPS Device

A non-differentially corrected GPS device (SPI Pro X, GPSports Systems, Australia) was worn in a harness between the scapulae, as per manufacturer's instructions. The device comprises a 5 Hz GPS microcontroller and a proprietary interpolation algorithm that outputs positional data at a 15 Hz frequency. The 15 Hz interpolation is suggested to enhance measurement accuracy compared to the raw 5 Hz data; however, technical specifications regarding this algorithm were unavailable and it is not possible to determine its effect on distance measurement validity. GPS devices do not directly measure distance; however, researchers and practitioners in the team sport domain are frequently interested in assessing distance rather than positional coordinates. Therefore terms related to ‘distance measurement’ are used throughout this paper, in preference to ‘distance calculation’, as they hold intuitive relevance in this field.

Raw 15 Hz GPS data were downloaded using the manufacturer's proprietary software (Team AMS v2.1), and exported for manual analysis. Data recorded outside the six exercise blocks were excluded from analyses. Distances were calculated within the proprietary software and no post processing was applied to raw GPS data. Precise velocity regulation allowed calculation of reference distances at the same frequency as the GPS data. The 15 Hz output frequency was expected to yield ≈80 000 observations per trial. Consistent with the aim of this study, additional variables calculated by the proprietary software (e.g. speed, acceleration, impact, body load) were not analysed.

### Data Analysis

Statistical analyses were performed using PASW (v18.0, SPSS Inc., USA). Measurement validity was considered to constitute accuracy and reliability, and a multifaceted statistical approach was implemented to assess these. Measurement accuracy was evaluated by calculating total, and within-epoch biases between GPS and reference distance measures. Two-tailed paired t-tests were performed to test the null hypothesis H_0_: distance_GPS_ = distance_reference_ for each protocol. These tests determined whether GPS measures differed systematically from reference distances; however, they cannot be used as the sole indicators of agreement [39]. Factorial analysis of variance was also performed to detect effects of trial (i.e. Shuttle and Curvilinear) and movement phase (i.e. Walk, Jog, Run and Sprint) on GPS distance measurement biases. That is, the null hypotheses H_0_: bias_curvilinear_ = bias_shuttle_, and H_0_: bias_walk_ = bias_jog_ = bias_run_ = bias_sprint_ were tested to determine whether GPS uncertainties were systematically affected by the presence of rapid directional change and/or differing movement velocities. Statistically significant interactions were explored using Bonferroni corrected paired comparisons. Previous data indicate 99 observations will provide 95% statistical power to detect a 5% difference between GPS and reference distance measures, assuming a standard deviation (SD) of 13.6% (Cohen's d = 0.37) [25]. Data were subjected to Levene's test for equality of error variances. Data displayed heteroscedastic error variance (Shuttle p = 0.001; Curvilinear p<0.001) and were logarithmically transformed and reanalysed. To aid interpretation data are reported in the unit of measurement, or relative to reference measures.

Relative measurement reliability was evaluated by calculating Pearson's correlation coefficients (r), regression coefficients (slope) and two-way random-effects intraclass correlation coefficients (ICC) [39], [40]. These statistics describe the reliability with which GPS distance increases as a function of reference distance, but are not indicative of agreement between measurement tools [41]. Absolute measurement reliability was evaluated by calculating the standard error of measurement 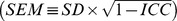, coefficient of variation 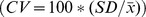 and 95% limits of agreement 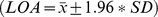 [39], [41]. These statistics indicate total measurement uncertainty (i.e. systematic+random error), and facilitate comparisons between experiments using different designs [42]. In-text data are reported as mean ± SD. Statistical significance for all calculations was set at α<0.05.

## Results

Satellite acquisition during curvilinear (8.19±1.82 satellites) and shuttle (8.44±1.57 satellites) trials was consistent with several previous studies evaluating GPS measurement validity [18], [19], [21], [23], [33].

### Measurement accuracy

Compared to the 13 200 m reference, distance was statistically significantly overestimated during the curvilinear trial (13 543.92±105.45 m; T_431 870_ = 772.40, p<0.001) and underestimated during the shuttle trial (12 780.68±325.61 m; T_460 226_ = −403.35, p<0.001). The between-trial bias was also statistically significant (F_1, 892 090_ = 386 116.42, p<0.001).

**Figure 2:**
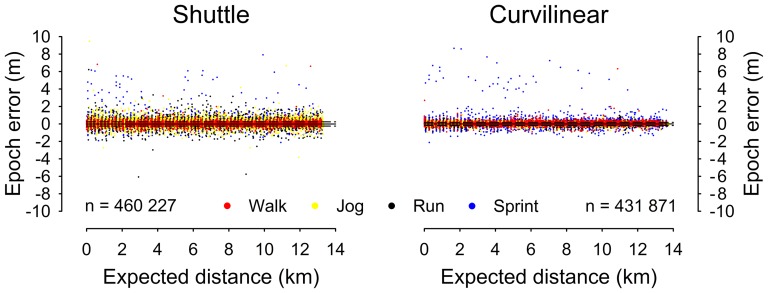
Within-epoch measurement uncertainty during shuttle and curvilinear trials. Solid reference lines = mean bias. Dashed reference lines = 95% limits of agreement. Differing sample sizes reflect discrepant mean sampling frequencies (Shuttle = 14.15±0.20 Hz; Curvilinear = 13.27±1.44 Hz), which were below the specified 15 Hz interpolation frequency.

Statistically significant effects of movement phase on measurement bias were detected during the curvilinear (F_3, 431 867_ = 31.23, p<0.001) and shuttle trials (F_3, 460 223_ = 15.42, p<0.001), although these differences were small (Table 1). Within-epoch biases during each movement phase are depicted graphically in Figure 2. The dispersion of within-epoch biases was widest during the shuttle trial. This effect was particularly evident during the Jog and Run phases, when bias dispersion approximated that of the sprint phase.

**Table 1:** Distance measurement biases during shuttle and curvilinear adaptations of the Loughborough Intermittent Shuttle-running Test.

Protocol	Total (%)	Walk (%)	Jog (%)	Run (%)	Sprint (%)
Shuttle	−2.16±3.84*	−2.18±4.23^j^ ^r^ ^s^	−2.20±3.42^w^ ^s^	−2.16±3.41^w^ ^s^	−1.92±3.63^w^ ^j^ ^r^
Curvilinear	2.99±2.96	2.99±4.06^j^ ^r^ ^s^	2.95±1.12^w^ ^s^	2.95±1.33^w^ ^s^	3.16±1.87^w^ ^j^ ^r^

Table reports mean (± s) within-epoch measurement biases relative to reference measures.

*Statistically significantly different to the curvilinear trial (p<0.001).

w j r s Statistically significantly different to the **W**alk, **J**og, **R**un or **S**print movement phases (p<0.001).

### Measurement Reliability

Table 2 summarises the relative and absolute reliability of GPS distance measures. GPS and reference distance were strongly correlated during curvilinear and shuttle trials (Table 2), indicating excellent relative measurement reliability. Moreover, regression coefficients approximated 1 during both trials (Table 2). Absolute reliability metrics (SEM, LOA and CV) were larger during the shuttle trial (Table 2) indicating superior absolute measurement reliability during the curvilinear protocol.

**Table 2:** GPS measurement reliability during shuttle and curvilinear adaptations of the Loughborough Intermittent Shuttle-running Test.

Protocol	r	Slope	ICC	SEM (m)	LOA (m)	CV (%)
Shuttle	0.99*	0.97	0.99*	119	±453	2.44
Curvilinear	1.00*	1.03	1.00*	0	±223	2.16

Table reports comparisons of GPS and reference distance measurement for Pearson's product-moment correlation (r), linear regression coefficient (Slope) intraclass correlation coefficient (ICC), standard error of measurement (SEM), Bland Altman's absolute limits of agreement (LOA), and the coefficient of variation (CV).

*p<0.001.

## Discussion

This study utilised a movement pattern that is representative of key aspects of high level team sport match-play to systematically evaluate the effect of rapid directional change on the distance measurement validity of a previously untested GPS device. The main finding is that rapid directional change degrades GPS distance measurement accuracy and absolute reliability, and this effect is independent of movement velocity.

The systematic positive bias between GPS and reference distances during the curvilinear trial is consistent with previous investigations employing running protocols with similarly low directional demands [18], [24]. Multidirectional running protocols are also associated with systematic distance measurement biases, but the magnitude and direction of uncertainty is inconsistent [16], [18], [25], [33]. The negative distance measurement bias during the shuttle trial is consistent with uncertainties reported during simulated court sports, team sports and non-linear running [18], [33]; however, positive biases have been reported during field hockey and team sport simulations [16], [25].

Consistent with the present results, the only previous study to evaluate the effect of multidirectional demands on GPS distance measurement validity reported positive and negative biases during linear and multidirectional running, respectively, with the largest absolute bias during non-linear running [18]. However, the self-selected velocity profiles differed between trials and it is unclear whether measurement validity was affected by differing directional demands, velocity demands, or a combination of these. Identical velocity regulation during the curvilinear and shuttle trials in the present study controlled for any effect of movement velocity on distance measurement validity. Thus the present findings indicate rapid multidirectional movement patterns degrade GPS distance measurement validity, and that this effect is independent of movement velocity.

Excellent relative measurement reliability during curvilinear and shuttle trials are consistent with previous investigations [16], [25], [32]. Nonetheless, larger absolute reliability metrics during the shuttle trial demonstrate reduced distance measurement validity during rapid multidirectional movement patterns.

The magnitude of shuttle trial distance measurement uncertainty was 3.17%. Uncertainties of similar magnitudes have previously underpinned support for the use of GPS technology during team sport-related activities [16], [18]–[25]. Indeed, when applied to a time-motion analysis of elite soccer match-play this uncertainty indicates match distance (10 627–12 027 m) may be underestimated by just 340–380 m, depending upon playing position [43]. In addition to the magnitude of uncertainty; however, it is also important to consider which movement patterns will be most affected by this inaccuracy. As rapid multidirectional movements are an important determinant of physiological load and match outcomes [28]–[31] the present results suggest GPS devices are likely to misrepresent some critical aspects of match-play. This has important implications for the way in which GPS technology is used in the team sport domain. As GPS measurement validity is also reduced during sprinting and rapid acceleration [16], [19], [22], [23], [32], [33] it appears GPS may not be an appropriate tool for evaluating match-play activity profiles, or monitoring athletes' physiological load. Given that iterative device development (i.e. newer hardware and software components) and faster sampling frequencies are proposed to improve GPS measurement validity [16], [19], [22], [33], this is particularly pertinent when utilising devices featuring older componentry and/or slower sampling frequencies.

Body lean angle is proposed to account for a substantial proportion of negative distance measurement bias during high speed nonlinear running [18], [44] and the proximal anatomical position of the present device predisposes it to similar uncertainty. This should be an important consideration when attempting to evaluate the criterion validity of GPS devices; however, post-hoc correction for lean angle would contrast the aim of this study by reducing its ecological validity and misrepresenting the measurement accuracy likely to be realised during real-world use. The effect of device position on measurement validity should be addressed during product development, when it can be balanced against other design constraints such as comfort, player safety and device exposure to impact.

The reduction in distance measurement validity during the shuttle trial may be explained by examining the interaction between movement demands and GPS position sampling. As intermittent GPS sampling partitions continuous movement paths into discrete linear segments, GPS devices are constrained to calculating cumulative device displacement across individual sampling epochs. While distance may be accurately quantified by displacement during linear movements, it will be underestimated when non-linear movements occur within a sampling epoch. Moreover, as rapid multidirectional movements will likely cause frequent separation between distance and displacement, the negative measurement uncertainty introduced by intermittent GPS sampling is likely to be largest during many of the most critical match-play activities.

While uncertainty induced by intermittent position sampling will affect all GPS devices, this mechanism may also explain the superior measurement validity of higher frequency devices during multidirectional running [19], [33]. Faster sampling frequencies increase the resolution with which continuous movement paths are partitioned into linear segments and, therefore, can be expected to reduce the magnitude of separation between device distance and displacement during non-linear movements. Nonetheless, sub-optimal distance measurement validity during the shuttle trial indicates the present device's 5 Hz sampling frequency and/or 15 Hz interpolation algorithm are insufficient to accurately quantify distance during rapid directional change. Further research is required to determine the optimal sampling frequency for quantifying performance during multidirectional movement patterns, such as those common to many team sports.

The proposed mechanism underlying distance measurement uncertainty cannot account for the positive distance bias recorded during curvilinear trials. This uncertainty is consistent with previous investigations [18], [24] yet its source remains unclear. Participant deviation outside the marked curvilinear running track, perhaps due to intentional protocol non-adherence or the neuromuscular fatigue induced by the LIST [45], would manifest positive measurement bias. However, adherence to marked running tracks and the precisely controlled velocity profile were monitored throughout all trials to preclude these confounds. Device-specific software components are proposed to affect measurement validity [16], but proprietary protection of these components makes evaluation of their contribution to measurement uncertainty problematic. Indeed, the contribution of the present device's 15 Hz interpolation algorithm to the observed measurement uncertainty remains unclear. Nonetheless, software components cannot account for the between-trial differences in measurement validity as device configuration remained identical throughout the study.

It is important to note that, although the LIST movement pattern is representative of several key aspects of match-play [35] and offers advantages for validating GPS devices compared to previous methodologies, it remains difficult to accurately simulate the complexity of match-play within a controlled environment. This limitation is common to all similar investigations, and should be considered when attempting to generalise these findings to match-play.

As multiple units of the present device were not simultaneously compared it remains unclear whether inter-unit variability will preclude interchangeable use of devices among multiple athletes, and caution should be taken when comparing data between devices.

This study used the movement pattern of a precisely controlled exercise protocol that is representative of several key aspects of soccer match-play to systematically assess the effect of rapid multidirectional movements on GPS distance measurement validity. Rapid directional change degrades GPS distance measurement validity, and this effect is independent of movement velocity. Caution is recommended when relying on GPS to quantify team sport athletes' performance as current device technology appears unable to accurately quantify movements that play a critical role in determining physiological load and competitive match outcomes.
